# Production of 1,2-propanediol from glycerol in *Klebsiella pneumoniae* GEM167 with flux enhancement of the oxidative pathway

**DOI:** 10.1186/s13068-023-02269-4

**Published:** 2023-02-06

**Authors:** Min-Ho Jo, Jung-Hyun Ju, Sun-Yeon Heo, Jaehoon Cho, Ki Jun Jeong, Min-Soo Kim, Chul-Ho Kim, Baek-Rock Oh

**Affiliations:** 1 Microbial Biotechnology Research Center, Jeonbuk Branch Institute, Korea Research Institute of Bioscience and Biotechnology (KRIBB), Jeongeup, Jeonbuk 56212 Republic of Korea; 2 Green and Sustainable Materials R&D Department, Korea Institute of Industrial Technology, Cheonan, Chungcheongnam 31056 Republic of Korea; 3 Department of Chemical and Biomolecular Engineering and Institute for the BioCentury, KAIST, Daejeon, 34141 Republic of Korea

**Keywords:** 1,2-propanediol, *Klebsiella pneumoniae*, Glycerol, Lactic acid, Oxidative pathway

## Abstract

**Background:**

To support the sustainability of biodiesel production, by-products, such as crude glycerol, should be converted into high-value chemical products. 1,2-propanediol (1,2-PDO) has been widely used as a building block in the chemical and pharmaceutical industries. Recently, the microbial bioconversion of lactic acid into 1,2-PDO is attracting attention to overcome limitations of previous biosynthetic pathways for production of 1,2-PDO. In this study, we examined the effect of genetic engineering, metabolic engineering, and control of bioprocess factors on the production of 1,2-PDO from lactic acid by *K. pneumoniae* GEM167 with flux enhancement of the oxidative pathway, using glycerol as carbon source.

**Results:**

We developed *K. pneumoniae* GEM167*ΔadhE*/pBR-1,2PDO, a novel bacterial strain that has blockage of ethanol biosynthesis and biosynthesized 1,2-PDO from lactic acid when glycerol is carbon source. Increasing the agitation speed from 200 to 400 rpm not only increased 1,2-PDO production by 2.24-fold to 731.0 ± 24.7 mg/L at 48 h but also increased the amount of a by-product, 2,3-butanediol. We attempted to inhibit 2,3-butanediol biosynthesis using the approaches of pH control and metabolic engineering. Control of pH at 7.0 successfully increased 1,2-PDO production (1016.5 ± 37.3 mg/L at 48 h), but the metabolic engineering approach was not successful. The plasmid in this strain maintained 100% stability for 72 h.

**Conclusions:**

This study is the first to report the biosynthesis of 1,2-PDO from lactic acid in *K. pneumoniae* when glycerol was carbon source. The 1,2-PDO production was enhanced by blocking the synthesis of 2,3-butanediol through pH control. Our results indicate that *K. pneumoniae* GEM167 has potential for the production of additional valuable chemical products from metabolites produced through oxidative pathways.

**Supplementary Information:**

The online version contains supplementary material available at 10.1186/s13068-023-02269-4.

## Background

Glycerol is the major by-product from the production of biodiesel and accounts for about 10% (w/w) of crude biodiesel [1]. This surplus of crude glycerol requires treatment before discharge into the environment [2]. Glycerol has been considered a low-cost feedstock for industrial value-added products because it is inexpensive and abundant [3], and it is mainly used for microbial production of 1,3-propanediol (1,3-PDO), ethanol, 2,3-butanediol (2,3-BDO), and lactic acid [4]. Glycerol generates higher yields of reduced metabolites (e.g., succinate, ethanol, 1,3-PDO) than glucose [5] because it produces twice the reducing equivalents per carbon when converted to glycolytic intermediates, such as phosphoenolpyruvate (PEP) or pyruvate [3].

1,2-propanediol (1,2-PDO), also known as propylene glycol, has been widely used as a building block in the chemical and pharmaceutical industries, monomer for the production of unsaturated polyester resins, and as a non-toxic replacement for ethylene glycol in deicers and antifreeze products [6, 7]. More than 1.36 million tons of racemic 1,2-PDO are produced worldwide every year. In 2020, the total 1,2-PDO produced worldwide had a value of approximately $373 million, and this total is expected to exceed $398 million by 2026, with a compound annual growth rate of 1.6%. Currently, most commercial production of 1,2-PDO is by chemical methods that require high pressure, high temperature, and non-catalytic hydrolysis of propylene oxide, and these methods produce racemic mixtures [7]. However, biologically based industrial production of chemicals is becoming increasingly attractive because of the need to use a wider range of biomass resources, the safer manufacturing processes, and the reduced environmental impact [8].

**Fig. 1:**
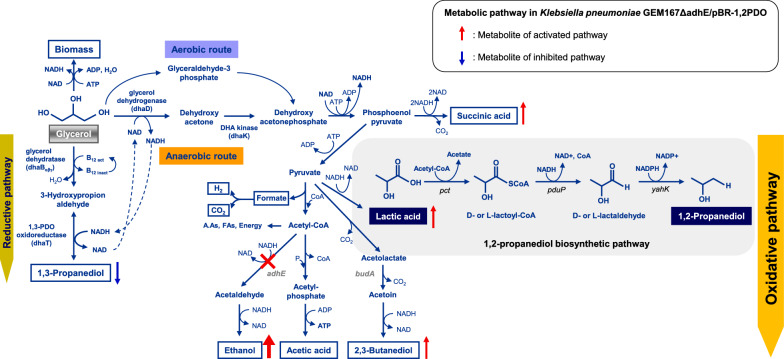
Pathway of 1,2-PDO biosynthesis from glycerol in *K. pneumoniae* GEM167*ΔadhE*/pBR-1,2PDO, with flux enhancement of the oxidative pathway

Microbial 1,2-PDO biosynthesis is mainly by two biosynthetic pathways: production of *S*-1,2-PDO through catabolism of 6-deoxyhexose from specific bacteria and yeast [9, 10] and production of *R*-1,2-PDO through the methylglyoxal pathway from glucose using *Clostridia* strains [11, 12]. Most current studies focus on the methylglyoxal pathway due to the limited availability of 6-deoxyhexose [13, 14]. For example, the production of 1,2-PDO from glycerol via the methylglyoxal pathway in *K. pneumoniae* has been reported [15]. However, it is difficult to achieve high concentrations and yields of 1,2-PDO using this method because methylglyoxal is cytotoxic, and sub-millimolar concentrations can cause cell death [16, 17]. To overcome this limitation, some recent research examined the microbial bioconversion of lactic acid into 1,2-PDO (Fig. 1). This artificial pathway for the biosynthesis of 1,2-PDO from glucose, with lactic acid as an intermediate, consists of two general steps. First, propionate CoA-transferase (*pct*) converts lactic acid into lactaldehyde, and then propanal dehydrogenase (*pduP*) and lactaldehyde reductase (*yahK*) sequentially reduce the lactaldehyde to 1,2-PDO [18, 19]. For these reasons, research on the production of 1,2-PDO from lactic acid is currently attracting attention. Although the 1,2-PDO titer obtained through the methylglyoxal pathway of *K. pneumoniae* was higher than that of all engineered strains that the pathway use methylglyoxal as an intermediate [15], engineered *E. coli* using lactic acid as an intermediate in the 1,2-PDO synthesis pathway produced higher 1,2-PDO titers [19]. Therefore, in this study, considering the limitations of 1,2-PDO production using the methylglyoxal pathway, we intend to biosynthesize 1,2-PDO from glycerol using an artificial pathway that biosynthesizes 1,2-PDO using *K. pneumoniae*.

*K. pneumoniae* can grow under aerobic or anaerobic conditions using glycerol as carbon source [20, 21]. Its specific growth rate is usually greater than 0.9 per h, even in glycerol minimal medium [20]. Another advantage of this species is that most of the genetic manipulation methods used for *Escherichia coli* can also be used in *K. pneumoniae* without significant modifications because they are evolutionarily related and biochemically similar [22, 23]. Although *K. pneumoniae* has been extensively studied, the biotechnological potential and physiological aspects of a strain have not yet been fully elucidated. Therefore, further studies of *K. pneumoniae* are necessary to confirm its potential for use in the chemical industry [4].

Previous studies of the metabolism of the *K. pneumoniae* GEM167 strain indicated it had decreased levels of metabolites related to the reduction of glycerol (1,3-PDO), but increased levels of metabolites related to the oxidation of glycerol (2,3-BDO, ethanol, lactic acid and succinate). The enhanced flux to the glycerol oxidation pathway in this strain suggested it has the potential for the efficient production of useful substances by when glycerol is a carbon source [24]. Therefore, we examined the effect of genetic engineering, metabolic engineering, and control of bioprocess factors on the production of 1,2-PDO from lactic acid by *K. pneumoniae* GEM167, with glycerol as carbon source.

## Results and discussion

### Construction of *K. pneumoniae* GEM167*ΔadhE* and measurement of 1,2-PDO production

*K. pneumoniae* GEM167 is a mutant strain that has an enhanced oxidation pathway. Thus, this strain has lower production of 1,3-PDO (a product of the reduction pathway) and higher production of ethanol (a product of the oxidation pathway) than the strain from which it was derived (*K. pneumoniae* ATCC 200721) [24]. We initially blocked ethanol biosynthesis in *K. pneumoniae* GEM167 by deletion of *adhE* (aldehyde dehydrogenase), so this strain could be further engineered used to produce 1,2-PDO **(**Fig. 1**)**.

We first compared the metabolites produced by two strains (GEM167 and GEM167*ΔadhE*) by use of flask culture for 12 h. The results indicated that the ethanol production was 0.1 ± 0.1 g/L in the GEM167*ΔadhE* strain and 7.0 ± 0.1 g/L in the GEM167 strain, demonstrating successful inhibition of ethanol biosynthesis **(**Table 1**)**.

**Table 1:** Metabolite analysis of two *K. pneumoniae* GEM167 strains after flask cultivation for 12 h

Strain	Metabolite concentration						
	Glycerol consumption (g/L)	2,3-BDO (g/L)	Lactic acid (g/L)	Acetic acid (g/L)	Succinate (g/L)	Ethanol (g/L)	1,3-PDO (g/L)
GEM167	19.1 ± 1.4	0.3 ± 0.1	1.4 ± 0.3	0.0	0.4 ± 0.1	7.0 ± 0.1	0.2 ± 0.1
GEM167 *ΔadhE*	3.9 ± 1.2	0.0	1.2 ± 0.1	1.3 ± 0.4	0.1 ± 0.1	0.1 ± 0.1	0.1 ± 0.1

To produce 1,2-PDO in *K. pneumoniae* GEM167*ΔadhE*, we expressed proteins encoded by the *pct*, *pduP*, and *yahK* genes using the lac promoter in the pBR322 plasmid with IPTG as an inducer (Fig. 1). Thus, lactic acid is first converted to lactoyl-CoA by *pct* (lactoyl-CoA transferase from *M. elsdenii*); lactoyl-CoA is converted to lactaldehyde by *pduP* (lactaldehyde dehydrogenase from *S. enterica*); and lactaldehyde is converted to 1,2-PDO by *yahK* (lactaldehyde reductase from *E. coli*). Fed-batch fermentation was performed to produce 1,2-PDO. The initial glycerol concentration was 20 g/L, and glycerol was fed to the bioreactor after the glycerol levels decreased below 5–10 g/L. Incubation of the engineered *K. pneumoniae* GEM167*ΔadhE*/pBR-1,2PDO in a 5-L jar for 48 h led to production of 326.9 ± 30.2 mg/L 1,2-PDO. The other major metabolites were lactic acid, 2,3-BDO, and acetic acid **(**Table 2**)**.

**Table 2:** Metabolite analysis of the *K. pneumoniae* GEM167*ΔadhE*/pBR-1,2PDO strain after fed-batch cultivation for 48 h

Strain	Metabolites concentration							
	Glycerol consumption (g/L)	1,2-PDO (mg/L)	2,3-BDO (g/L)	Lactic acid (g/L)	Acetic acid (g/L)	Succinate (g/L)	Ethanol (g/L)	1,3-PDO (g/L)
GEM167*ΔadhE*/pBR-1,2PDO	31.3 ± 2.1	326.9 ± 30.2	4.7 ± 1.3	17.9 ± 0.7	2.4 ± 0.2	0.8 ± 0.1	1.8 ± 0.1	0.2 ± 0.1

The wild-type strains of some species mainly produce 1,2-PDO using *L*-rhamnose, *L*-fucose, glucose, xylose, mannose, and cellobiose as substrates [12, 25, 26]. Glucose or glycerol is generally used as substrates for 1,2-PDO production through the methylglyoxal pathway in engineered bacteria. Studies of 1,2-PDO production through the lactic acid pathway have only used glucose as a substrate, and only used genetically engineered *E. coli* [27]. To our knowledge, the present study is the first to report the biosynthesis of 1,2-PDO from lactic acid in *K. pneumoniae* with the use of glycerol as the substrate. Our results indicated that the metabolic engineering of the GEM167 mutant strain described here, which has an enhanced oxidation pathway, has great potential for the production of value-added materials from metabolic derivatives of the oxidation pathway.

### Effect of agitation speed on 1,2-PDO production by *K. pneumoniae* GEM167*ΔadhE*/pBR-1,2PDO

**Fig. 2:**
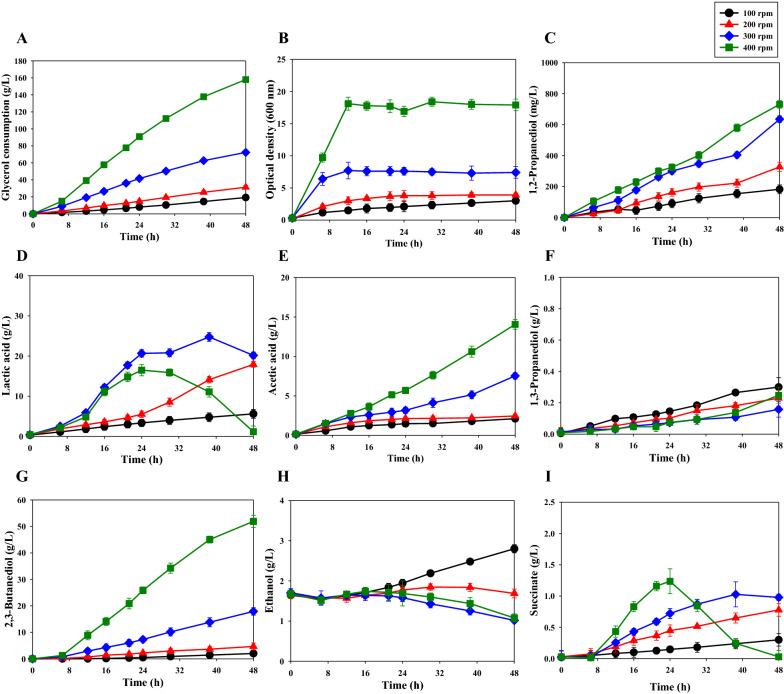
Effect of agitation speed on the cell growth and metabolite production by *K. pneumoniae* GEM167*ΔadhE*/pBR-1,2PDO. **A** glycerol consumption; **B** growth (OD_600 nm_); **C** 1,2-PDO concentration; **D** lactic acid concentration; **E** acetic acid concentration; **F** 1,3-PDO concentration; **G** 2,3-BDO concentration; **H** ethanol concentration; **I** succinate concentration. Cultivation was in a 5-L jar fermenter (37 °C, 0.5 vvm, pH 6 maintained using 28% v/v NH_4_OH) for 48 h with glycerol as carbon source. Black circles, 100 rpm; red triangles, 200 rpm; blue diamonds, 300 rpm; green squares, 400 rpm. Here and below: means ± SDs of triplicate measurements are shown. SD, standard deviation

We investigated the effect of agitation speed (100, 200, 300, 400 rpm) on 1,2-PDO production by *K. pneumoniae* GEM167*ΔadhE*/pBR-1,2PDO by growing cultures in 5-L jar fermenters for 48 h. The culture conditions were 37 °C, aeration at 0.5 vvm, and pH 6. The results show that the highest production of 1,2-PDO was 731.0 ± 24.7 mg/L at 400 rpm (Fig. 2C, Table 3). The glycerol consumption (158.0 ± 1.1 g/L) (Fig. 2A) and growth (OD_600nm_: 18.4 ± 0.7) (Fig. 2B) were also greatest at 400 rpm. The maximum lactic acid production (24.7 ± 1.1 g/L) was at 300 rpm, and this amount gradually decreased toward the end of culture (Fig. 2D). A previous study also reported that lactic acid or succinate increased and then decreased during *K. pneumoniae* culture [28]. It is also noteworthy that the acetic acid production increased sharply from 7.5 ± 0.4 g/L at 300 rpm to 14.1 ± 0.6 g/L at 400 rpm (Fig. 2E). Agitation speed also had a marked effect on the production of 2,3-BDO, with an increase from 4.7 ± 1.3 g/L at 200 rpm to 51.9 ± 2.3 g/L at 400 rpm (Fig. 2G). We did not use a higher agitation speed because there was excessive foaming during fermentation at 500 rpm (Additional file 1: Fig. S1).

**Table 3:** Effect of agitation speed on the metabolites of the *K. pneumoniae* GEM167*ΔadhE*/pBR-1,2PDO strain after fed-batch cultivation for 48 h

Agitation speed (rpm)	Maximum metabolite concentration								
	Glycerol consumption (g/L)	Cell growth (OD _600 nm_)	1,2-PDO (mg/L)	2,3-BDO (g/L)	Lactic acid (g/L)	Acetic acid (g/L)	Succinate (g/L)	Ethanol (g/L)	1,3-PDO (g/L)
100	19.2 ± 1.3	3.0 ± 0.4	182.4 ± 28.1	2.0 ± 2.1	5.6 ± 1.1	2.1 ± 0.3	0.3 ± 0.1	2.8 ± 0.1	0.3 ± 0.1
200	31.3 ± 2.1	3.9 ± 0.3	326.9 ± 30.2	4.7 ± 1.3	17.9 ± 0.7	2.4 ± 0.2	0.8 ± 0.1	1.8 ± 0.1	0.2 ± 0.1
300	72.3 ± 2.7	7.7 ± 1.3	635.3 ± 21.4	17.9 ± 1.4	24.7 ± 1.1	7.5 ± 0.4	1.0 ± 0.2	1.7 ± 0.3	0.2 ± 0.1
400	158.0 ± 1.1	18.4 ± 0.7	731.0 ± 24.7	51.9 ± 2.3	16.5 ± 1.4	14.1 ± 0.6	1.2 ± 0.2	1.7 ± 0.1	0.2 ± 0.1

The aeration increased as the agitation speed increased, and this led to increased glycerol consumption and cell growth. Previous research reported that many factors affected the culture process of *K. pneumoniae*, and that media composition and aeration had substantial effects, but were not crucial determinants [29]. The presence of oxygen inhibits the metabolism of glycerol to acetic acid and ethanol and increases the synthesis of lactic acid and 2,3-BDO, thereby increasing the demand for reducing equivalents and increased energy [30]. We confirmed that lactic acid and 2,3-BDO increased rapidly as aeration (agitation speed) increased. However, we observed that acetic acid also increased significantly as the agitation speed increased to 300 rpm and 400 rpm (Fig. 2E).

Intensive aeration leads to a significant increase in 2,3-BDO and acetic acid production. In the comparison of the molar conversion rate (mole of 2,3-BDO product/mole of consumed glycerol) according to agitation speed, the more the aerobic conditions became, the more the metabolic flux was shifted toward production 2,3-BDO (molar conversion of glycerol to 2,3-BDO, from 10.6% at 100 rpm to 33.6% at 400 rpm).

We examined the use of two approaches to resolve the problem of excessive production of 2,3-BDO. The first approach was a genetic manipulation that removed *budA* (a gene that functions in 2,3-BDO biosynthesis in *K. pneumoniae* GEM167*ΔadhE*) using metabolic engineering (Fig. 1). The second approach was pH control to suppress 2,3-BDO production as a bioprocess control factor.

### Use of metabolic engineering to increasing 1,2-PDO production and decrease 2,3-BDO production

Among the metabolites produced by *K. pneumoniae* GEM167*ΔadhE*/pBR-1,2PDO, 2,3-BDO was the major by-product. To enhance 1,2-PDO production, we removed *budA* to suppress 2,3-BDO biosynthesis. Previous studies of the genes involved in 2,3-BDO biosynthesis (*budB*, acetolactate synthase, conversion of pyruvate to acetolactate; *budA*, acetolactate decarboxylase, conversion of acetolactate to acetoin; and *budC*, acetoin reductase, conversion of acetoin to 2,3-BDO) indicated that blockage of *budA* effectively suppressed 2,3-BDO biosynthesis [31].

**Fig. 3:**
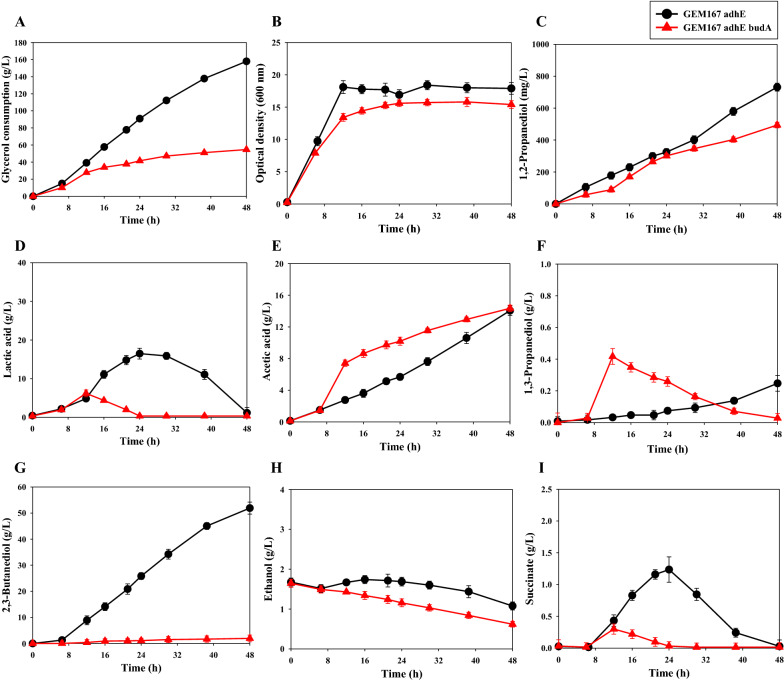
Effect of knockout of the *budA* gene on the cell growth and metabolite production by *K. pneumoniae* GEM167*ΔadhE*/pBR-1,2PDO. **A** glycerol consumption; **B** growth (OD_600 nm_); **C** 1,2-PDO concentration; **D** lactic acid concentration; **E** acetic acid concentration; **F** 1,3-PDO concentration; **G** 2,3-BDO concentration; **H** ethanol concentration; **I** succinate concentration. Cultivation of different strains was in a 5-L jar fermenter (37 °C, 400 rpm, 0.5 vvm, pH 6 maintained using 28% v/v NH_4_OH) for 48 h with glycerol as carbon source. Black circles, *K. pneumoniae* GEM167*ΔadhE*/pBR-1,2PDO strain; red triangles, *K. pneumoniae* GEM167*ΔadhEΔbudA*/pBR-1,2PDO strain

Thus, we compared 1,2-PDO production by *K. pneumoniae* GEM167*ΔadhE*/pBR-1,2PDO and *K. pneumoniae* GEM167*ΔadhEΔbudA*/pBR-1,2PDO (Fig. 3 and Table 4). After 48 h, *K. pneumoniae* GEM167*ΔadhEΔbudA*/pBR-1,2PDO produced only 1.9 ± 1.3 g/L of 2,3-BDO, indicating successful suppression of this pathway (Fig. 3G). However, this strain also had slightly decreased 1,2-PDO production (493.8 ± 17.9 mg/L) (Fig. 3C), but deletion of *budA* increased the carbon g/g yield of 1,2-PDO (Table 4). And this strain had greatly decreased lactic acid production (6.2 ± 0.9 g/L), although the two strains produced similar levels of acetic acid. Also, *K. pneumoniae* GEM167*ΔadhEΔbudA*/pBR-1,2PDO had greatly reduced glycerol consumption (54.7 ± 1.4 g/L) (Fig. 3A) and lower cell growth (OD_600nm_: 15.8 ± 0.7) (Fig. 3B), indicating that deletion of *budA* led to decreased cell growth.

**Table 4:** Metabolite analysis of the *K. pneumoniae* GEM167*ΔadhEΔbudA*/pBR-1,2PDO strain after fed-batch cultivation for 48 h

Strain	Maximum metabolite concentration								
	Glycerol consumption (g/L)	Cell growth (OD _600 nm_)	1,2-PDO (mg/L)	2,3-BDO (g/L)	Lactic acid (g/L)	Acetic acid (g/L)	Succinate (g/L)	Ethanol (g/L)	1,3-PDO (g/L)
GEM167*ΔadhE*/pBR-1,2PDO	158.0 ± 1.1	18.4 ± 0.7	731.0 ± 24.7	51.9 ± 2.3	16.5 ± 1.4	14.1 ± 0.6	1.2 ± 0.2	1.7 ± 0.1	0.2 ± 0.1
GEM167*ΔadhEΔbudA*/pBR-1,2PDO	54.7 ± 1.4	15.8 ± 0.7	493.8 ± 17.9	1.9 ± 1.3	6.2 ± 0.9	14.3 ± 0.4	0.3 ± 0.1	1.6 ± 0.1	0.4 ± 0.1

The two pyruvates that enter the 2,3-BDO biosynthetic pathway consume reducing equivalents during conversion to 2,3-BDO, and this plays an important role in regulating the intracellular NADH/NAD^+^ ratio [32]. These two nicotinamide adenine dinucleotides (NADH and NAD^+^) function as cofactors in more than 300 enzymes related to oxidation and reduction [33, 34]. Thus, the NADH/NAD^+^ ratio has a decisive effect on the intracellular redox balance and maintenance of the general conditions in which microorganisms can metabolize and grow [35].

The NADH/NAD^+^ ratio can be regulated by altering metabolic pathways that compete for NADH or NAD^+^ [36], and restriction of competing metabolic pathways increases the NADH/NAD^+^ ratio, inhibiting cell growth and glycolysis, slowing glycerol consumption [37]. Cell growth is associated with biosynthesis of NADH, but accumulation of NADH is not favorable for cellular material synthesis. In addition to the effect of redox balance on cell growth, there is also evidence that ATP production through acetic acid synthesis contributes to biomass synthesis, although increased acetic acid production can also inhibit cell growth. Because acetic acid is more toxic than the other products (1,3-PDO, ethanol, lactic acid), previous research examined the effect of acetic acid on growth of *K. pneumoniae* [38]. Consistent with previous studies, we found that deletion of *budA* increased the level of acetate. There is evidence that the accumulation of acidic metabolites can lead to defects in cell growth [31]. This is because 2,3-BDO biosynthesis in microorganisms helps control intracellular acidification by converting acids into neutral metabolites [39]. A previous study also reported inhibition of cell growth by knockout of *budA* in *Klebsiella oxytoca* [40].

We found that the level of acetic acid gradually increased in *K. pneumoniae* GEM167*ΔadhE*/pBR-1,2PDO. But cell growth and acetic acid production were rapid during the early stage of cell growth in *K. pneumoniae* GEM167*ΔadhEΔbudA*/pBR-1,2PDO. The slowing of cell growth in this strain after about 12 h may be related to the over-production of acetic acid (Fig. 3B and E).

Simple stoichiometry indicates that blockage of ethanol biosynthesis generates 2 extra moles of NADH per mole of acetyl-CoA and blockage of 2,3-BDO biosynthesis generates 1 extra mole of NADH per mole of acetyl-CoA. Thus, the surplus NADH generated by removal of *budA* may not have been properly consumed by these cells. This could lead to an excessive NADH/NAD^+^ ratio and disruption of the redox balance, which could adversely affect cell growth and 1,2-PDO production, as well as overall cell metabolism. Therefore, further metabolic engineering may be able to achieve a better redox balance in this strain by promoting consumption of the surplus reducing power and thereby increase 1,2-PDO production.

### Increasing 1,2-PDO production and decreasing 2,3-BDO production using pH as a bioprocess factor

**Fig. 4:**
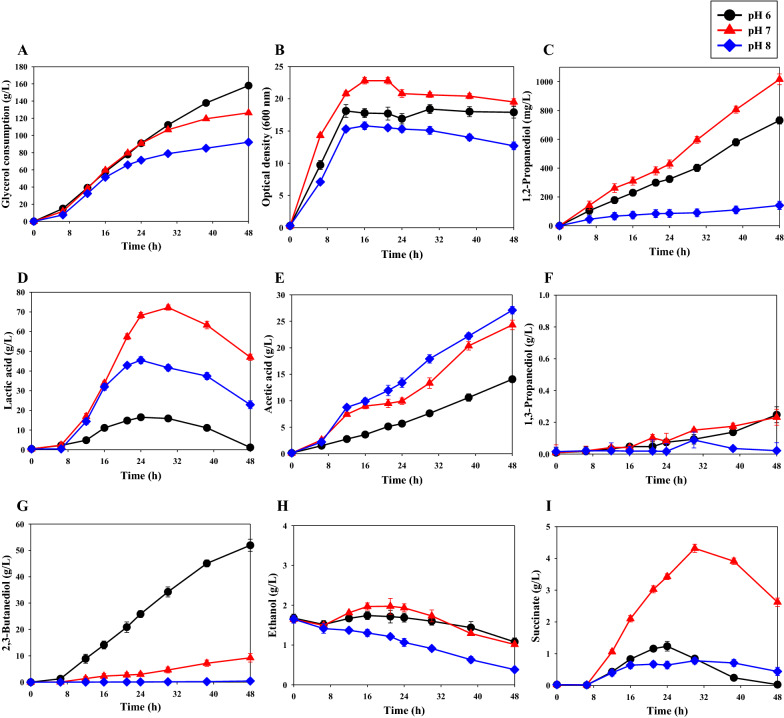
Effect of pH on the cell growth and metabolite production by *K. pneumoniae* GEM167*ΔadhE*/pBR-1,2PDO. **A** glycerol consumption; **B** growth (OD_600 nm_); **C** 1,2-PDO concentration; **D** lactic acid concentration; **E** acetic acid concentration; **F** 1,3-PDO concentration; **G** 2,3-BDO concentration; **H** ethanol concentration; **I** succinate concentration. Cultivation at 3 pH values was in a 5-L jar fermenter (37 °C, 400 rpm, and 0.5 vvm) for 48 h with glycerol as carbon source. Black circles, pH 6; red triangles, pH 7; blue diamonds, pH 8

We examined control of pH as the second approach to suppressing 2,3-BDO production and increasing 1,2-BDO production in *K. pneumoniae* GEM167*ΔadhE*/pBR-1,2PDO. A previous study showed that the acid-resistant strain *K. pneumoniae* G31 produced maximal 2,3-BDO at pH 5.4 [29], suggesting that pH can significantly affect 2,3-BDO synthesis. Therefore, we investigated the effect of pH on the production of 2,3-BDO and 1,2-PDO by *K. pneumoniae* GEM167*ΔadhE*/pBR-1,2PDO (Fig. 4 and Table 5). Initial experiments indicated that these cells did not grow well at pH 5, so we performed experiments in the range of pH 6 to pH 8. The results indicated that pH had a dramatic effect on production of 2,3-BDO and 1,2-BDO. The highest 1,2-PDO production was 1016.5 ± 37.3 mg/L at pH 7 (Fig. 4C). Glycerol consumption also decreased as pH increased (Fig. 4A). Cell growth was also greatest at pH 7 (OD_600nm_: 22.8 ± 0.5) (Fig. 4B). Notably, the maximum production of succinic acid was also at pH 7 (4.3 ± 0.1 g/L) (Fig. 4I).

**Table 5:** Metabolite analysis of the *K. pneumoniae* GEM167*ΔadhE*/pBR-1,2PDO strain after fed-batch cultivation at different pH values for 48 h

pH	Maximum metabolite concentrations								
	Glycerol Consumption (g/L)	Cell growth (OD _600 nm_)	1,2-PDO (mg/L)	2,3-BDO (g/L)	Lactic acid (g/L)	Acetic acid (g/L)	Succinate (g/L)	Ethanol (g/L)	1,3-PDO (g/L)
pH 6	158.0 ± 1.1	18.4 ± 0.7	731.0 ± 24.7	51.9 ± 2.3	16.5 ± 1.4	14.1 ± 0.6	1.2 ± 0.2	1.7 ± 0.1	0.2 ± 0.1
pH 7	126.3 ± 1.3	22.8 ± 0.5	1016.5 ± 37.3	9.2 ± 1.6	72.2 ± 1.3	24.3 ± 0.9	4.3 ± 0.1	2.0 ± 0.2	0.2 ± 0.1
pH 8	92.2 ± 1.2	15.8 ± 0.6	141.2 ± 29.1	0.4 ± 0.2	45.5 ± 1.8	27.1 ± 0.7	0.8 ± 0.1	1.6 ± 0.1	0.1 ± 0.1

We found that as the pH increased, the 2,3-BDO production decreased significantly, and production was negligible at pH 8 (Fig. 4G). However, the highest 1,2-PDO production was at pH 7 (1016.5 ± 37.3 mg/L) (Fig. 4C). Thus, the effect of pH on 1,2-PDO production is not entirely attributable to the reduced synthesis of 2,3-BDO.

One mole of NADH is required for synthesis of 1 mol of 2,3-BDO, but inhibition of 2,3-BDO synthesis leads to a surplus of NADH. This NADH could be used as a coenzyme for the biosynthesis of other metabolites or in the engineered 1,2-PDO pathway. Changes in the pH of the medium can cause metabolic shift [41]. NAD plays a central role in metabolism by functioning as a cofactor in redox reactions. The NADH/NAD^+^ cofactor pair has a regulatory effect on the expression of some genes and the activity of certain enzymes, and an excess NADH can disrupt the redox balance [42]. The production of 2,3-BDO was inhibited at pH 7 and pH 8, but 1,2-PDO synthesis was higher at pH 7 (Fig. 4C and G), which suggests the redox balance was more stably maintained at pH 7. This might be because these cells can maintain the redox balance by efficient use of the reducing power from excess NADH generated by inhibiting 2,3-BDO biosynthesis at pH 7, but cannot efficiently use the large excess of NADH generated at pH 8. In agreement, lactic acid production was higher at pH 7 (72.2 ± 1.3 g/L) than at pH 8 (45.5 ± 1.8 g/L at pH 8), and succinic acid production (which also requires NADH) was also significantly greater at pH 7 (4.3 ± 0.1 g/L) than at pH 8 (0.8 ± 0.1 g/L at pH 8). In addition, the pH of a medium affects cell growth and fermentation rates, as well as the yield and purity of metabolites [41]. And it was found the pH 7 was most favorable for cell growth in *K. pneumoniae* GEM167*ΔadhE*/pBR-1,2PDO. In agreement, the optimal range for growth of other *Klebsiella* strains is pH 6 to pH 8 [43].

Although cell growth was greater at pH 7 than at pH 6, it was lowest at pH 8 (Fig. 4B). Microorganisms adapt to the external environment by altering their metabolism, and changes in the flux of different metabolic pathways affect the amounts of metabolites produced and also affect cell growth. We suggest that pH may have affected 1,2-PDO production due to changes in the redox balance from alterations in the flux of different pathways. We also found that inhibition of 2,3-BDO biosynthesis by adjustment of pH (a bioprocessing factor) was more effective in increasing the level of 1,2-PDO than metabolic engineering. It is possible that extreme inhibition of 2,3-BDO biosynthesis achieved by metabolic engineering led to an extreme breakdown of the redox balance. On the other hand, pH control only moderately inhibited the biosynthesis of 2,3-BDO, so the alteration of the redox balance was less severe.

However, our use of pH control as a bioprocess factor still led to a relatively low yield of 1,2-PDO. The biosynthetic pathway of 1,2-PDO requires NAD(P)H. In view of this, the cause of the inadequate 1,2-PDO production—even at pH 7—is probably because the excess NADH was not efficiently used for 1,2-PDO biosynthesis. Ideally, more of the reducing power generated when controlling the flux of the metabolic pathways in this strain should be used to stimulate the 1,2-PDO pathway. Therefore, future metabolic engineering studies should examine methods that improve 1,2-PDO production by efficiently using the surplus reducing power to increase 1,2-PDO biosynthesis.

### Genetic stability of the 1,2-PDO production trait

We also examined the stability of the plasmid (pBR-1,2PDO) in the *K. pneumoniae* GEM167*ΔadhE*/pBR-1,2PDO strain. Thus, we grew the cells in fed-batch fermentation for 72 h and then collected them for cultivation on LB plates without antibiotic. We then tested colonies for maintenance of plasmid DNA by replica plating on medium with and without antibiotic. These experiments showed that pBR-1,2PDO was maintained at 100%, indicating excellent genetic stability [44].

## Conclusion

We developed *K. pneumoniae* GEM167*ΔadhE*/pBR-1,2PDO, which has blocked ethanol synthesis and engineered synthesis of 1,2-PDO. This strain produced of 1,2-PDO from lactic acid when glycerol was carbon source. Increasing the rate of agitation during growth further not only increased the production of 1,2-PDO but also increased the production of 2,3-BDO, a by-product. Control of pH effectively inhibited 2,3-BDO production and increased 1,2-PDO production, possibly because it allowed the cells to gradually adapt to their environment. This was the first study to biosynthesize 1,2-PDO from lactic acid in *K. pneumoniae* using glycerol as carbon source. This strain of *K. pneumoniae* GEM167, which has enhanced oxidative flux, has the potential for producing additional valuable materials derived from metabolites produced in the oxidative pathway.

## Materials and methods

### Bacterial strains, plasmids, and media

The bacterial strains and plasmids used in this study are listed in Table 6, and the primers used for PCR are listed (Additional file 1: Table S1**)**. The *K. pneumoniae* mutant strain GEM167 was derived from *K. pneumoniae* ATCC 200721 and described in a previous study [24].

**Table 6:** Strains and plasmids

Strain or plasmid	Relevant genotype and description	Source
Strains		
* E. coli* DH5α	Host of plasmid	Lab stock
* K. pneumoniae* GEM167		[24]
* K. pneumoniae ΔadhE*		This study
* K. pneumoniae ΔadhE ΔbudA*		This study
Plasmid		
pBR-1,2PDO	Lac promoter, pBR322 carrying *pduP-pct-yahK*, Tet	This study

*Escherichia coli* DH5α was used for DNA manipulation. Lambda-Red and FLP recombinases were expressed by helper plasmids pKD46 [45] and pCP20 [46], respectively. Replication of these plasmids is temperature sensitive, thus facilitating elimination. The pIJ773 vector was the source of the apramycin resistance gene. Plasmid pGEM-T Easy (Promega, Madison, WI, USA) and the pBHA vector (Bioneer, Daejeon, South Korea) were used for cloning. The pBR-1,2PDO plasmid contains genes in the 1,2-PDO biosynthetic pathway (Fig. 1): *pduP* (CoA-dependent lactaldehyde dehydrogenase or CoA-dependent propanal dehydrogenase from *Salmonella enterica*), *pct* (lactoyl-CoA transferase or propionate CoA-transferase from *Megasphaera elsdenii*), and *yahK* (lactaldehyde reductase or aldehyde reductase from *E. coli*).

Microbial cells were grown in LB medium (yeast extract [Difco], 0.5% [w/v]; Bacto-tryptone [Difco], 1.0% [w/v]; and NaCl, 1.0% [w/v]), or germ medium [47], supplemented with appropriate antibiotics (ampicillin [100 μg/mL] and/or apramycin [50 μg/mL] or tetracycline [10–50 μg/mL]). Germ medium at the flask scale contained 30 g/L glycerol, 1 g/L yeast extract, 2 g/L (NH_4_)_2_SO_4_, 10.7 g/L K_2_HPO_4_, and 5.24 g/L KH_2_PO_4_. Germ medium at the 5-L fementor scale contained 20 g/L glycerol, 1 g/L yeast extract, 2 g/L (NH_4_)_2_SO_4_, 10.7 g/L K_2_HPO_4_, and 5.24 g/L KH_2_PO_4_. The following compounds were added to all germ media: 0.2 g/L MgSO_4_, 0.02 g/L CaCl_2_·2H_2_O, 1 mL Fe solution (5 g/L FeSO_4_·7H_2_O and 4 mL HCl [37%, w/v]), 1 mL trace element solution (70 mg/L ZnCl_2_, 100 mg/L MnCl_2_·4H_2_O, 60 mg/L H_3_BO_3_, 200 mg/L CoCl_2_·4H_2_O, 20 mg/L CuCl_2_·2H_2_O, 25 mg/L NiCl_2_·6H_2_O, 35 mg/L Na_2_MoO_4_·2H_2_O, and 4 mL HCl [37%, w/v]), and 10 μg/mL tetracycline.

### Construction of recombinant plasmids

The strategy used to construct pBR-1,2PDO is presented (Additional file 1: Fig. S2) The *pduP*, *pct*, and *yahK* genes were synthesized by Bioneer Co. Ltd. (Korea). These sequences were cloned into the pBHA vector (Bioneer, Daejeon, South Korea), followed by nucleotide sequencing to confirm there were no errors introduced during cloning. A *Nhe*I-*Spe*I fragment containing the *pct* gene was inserted into the corresponding restriction sites downstream of the *pduP* sequence (pBHA-pdup). pBHA-*pduP-pct-yahK* was generated by sequential insertion of *Nhe*I-*Spe*I fragments containing *pct* (pBHA-pct) and *yahK* (pBHA-yahk) into the *Spe*I sites of pBHA-pduP. The lacZ promoter sequence (P_lacZ_) was synthesized by Bioneer Co. Ltd. (Korea). These DNA fragments were cloned into the pGEM-T Easy vector, followed by nucleotide sequencing to confirm there were no errors introduced during cloning. pGEM-P_lacz_-*pduP*-*pct*-*yahK* was obtained by inserting the *Nhe*I-*Spe*I fragment (containing *pduP*-*pct*-*yahK* from pBHA-*pduP*-*pct*-*yahK*) into the *Spe*I sites of pGEM-PlacZ. Finally, pBR322 was cleaved with *Sca*I, treated with alkaline phosphatase, ligated with a DNA fragment obtained by digestion of pGEM-PlacZ-*pduP*-*pct*-*yahK* with *Not*I, and then treated with the Klenow fragment to yield plasmid pBR-1,2PDO (*pduP*-*pct*-*yahK*). Electroporation was used to transform the final plasmid into *K. pneumoniae* [48].

### Gene deletion

#### Knocking out adhE to block ethanol biosynthesis

The strategy used to knock out *adhE* gene is presented (Additional file 1: Fig. S3). To delete the chromosomal *adhE* gene (aldehyde dehydrogenase), 0.65-kb DNA sequences upstream and downstream of *adhE* were amplified by PCR using oligo-nucleotides P1 (5ʹ-TCC GCA GCA TCA TCA AAA TTG GCG-3ʹ) and P2 (5ʹ-ACC GGA GCA ACT TCG GCT TTC *GAT ATC* ATT CGA GCA TCT GCA GCG GC-3ʹ; bases in italics indicate the *Eco*RV site) which bind to the upstream region and P3 (5ʹ-GCC GCT GCA GAT GCT CGA AT*G ATA TC*G AAA GCC GAA GTT GCT CCG GT-3ʹ; bases in italics indicate the *Eco*RV site) and P4 (5ʹ-TGT ATA ATC CAC AGA CCT CGT TA-3ʹ) which bind to the downstream region. The PCR conditions were as follows: initial denaturation at 95 ℃ for 5 min; 30 cycles at 95 ℃ for 30 s, 55 ℃ for 30 s, and 72 ℃ for 90 s; and extension at 72 ℃ for 7 min. The PCR products were annealed using primers P1 and P4 and the resultant product cloned into pGEM-T Easy. After treatment with the Klenow fragment, an apramycin resistance gene (*aac(3)IV*, obtained from pIJ773 by digestion with *Eco*RI and *Hind*III) was inserted into the *Eco*RV site of the PCR product generated above. The resultant plasmid, pT-*adhE*-*Apra*, was used as a template for PCR amplification of the deletion cassette, which was then introduced into *K. pneumoniae* GEM167 by electroporation [49], followed by homologous recombination to create a chromosomal mutant. Correct integration of the DNA fragment was confirmed by Southern hybridization using regions upstream of *adhE* and *aac(3)IV* to probe KpnI-digested chromosomal DNA with probes that were labeled with digoxigenin-dUTP (Roche Diagnostics GmbH, Mannheim, Germany) [50].

#### Knocking out budA to block 2,3-BDO biosynthesis

The strategy used to knock out *budA* gene is presented (Additional file 1: Fig. S4). The chromosomal acetolactate decarboxylase gene (*budA*), which functions in 2,3-BD biosynthesis, was deleted by first amplifying 0.3-kb DNA sequences upstream and downstream by PCR using the primer pairs P1 (5ʹ-ATC GAA AAC GTC TCA AAC CAG C-3ʹ) and P2 (5ʹ-GAT CGT CGA GGA CGT CGG TC*G TTA AC*A TAG ACC TGA CTG CTG AAG G-3ʹ) for the upstream region and P3 (5ʹ-CCT TCA GCA GTC AGG TCT ATG TTA ACG ACC GAC GTC CTC GAC GAT C-3ʹ) and P4 (5ʹ-CCT TAA CTT TCT ACG GAA CGG A-3ʹ) for the downstream region (bases in italics indicate the *Hpa*I site). The PCR conditions were as follows: initial denaturation at 95 ℃ for 5 min; 30 cycles of 95 ℃ for 30 s, 55 ℃ for 30 s and 72 ℃ for 90 s; and extension at 72 ℃ for 7 min. The PCR products were annealed using P1 and P4, and the resultant product was cloned into pGEM-T Easy. After treatment with the Klenow fragment, an apramycin resistance gene (*aac(3)IV*, obtained from pIJ773 by digestion with *Eco*RI and *Hind*III) was inserted into the *Hpa*I site of the PCR product. The resultant plasmid, pT-budA-Apra, was used as a template for PCR amplification of the deletion cassette, which was then introduced into *K. pneumoniae* GEM167*ΔadhE* by electroporation [48]. Correct integration of the DNA fragment by homologous recombination was confirmed by Southern hybridization using the upstream regions of *budA* and *aac(3)IV* to probe *Pst*I-digested chromosomal DNA with probes that were labeled with the digoxigenin-dUTP system (Roche Diagnostics GmbH, Mannheim, Germany) [44].

### Fermentation by recombinant *K. pneumoniae* strains

Seed cells for fermentation were prepared in 10 mL of LB medium that was in a 50 mL conical tube. Seed cultures were incubated on a shaker (37 ℃ with agitation at 200 rpm for 9 h) and then inoculated into 250 mL of medium containing tetracycline (10 μg/mL) that was in a 1-L round flask. These flasks were then incubated (37 ℃ with agitation at 200 rpm for 8 h) and then inoculated into a 5-L jar fermenter at 10% (v/v). Fed-batch fermentations (37 ℃ with agitation at 200 rpm and aeration at 0.5 vvm for 48 h) were performed in the 5-L vessel (Kobiotech. Co., Ltd, Korea) that contained 2.5 L of fermentation medium with tetracycline (10 μg/mL). The pH was controlled by automatic addition of 28% (v/v) NH_4_OH. For fed-batch fermentation, a pulse of glycerol was added to the medium, and it was fed when the concentration of glycerol in the fermentation broth decreased to 5–10 g/L. The carbon source was pure glycerol (purity 99%, w/w) [31]. Isopropyl β-D-1-thiogalactopyranoside (IPTG) was added to the culture medium as an inducer (final concentration: 0.5 mM).

### Analytical methods

Optical density at 600 nm (OD_600nm_) was measured to monitor cell growth. Culture broth concentrations of metabolites (glycerol, lactic acid, acetic acid, succinate, 1,3-PDO, 2,3-BDO, and ethanol) were determined using a high-performance liquid chromatography (HPLC) system (Agilent 1200 series, Agilent Technologies, Santa Clara, CA, USA) that was equipped with a refractive index detector and an organic acid analysis column (300 × 7.8 mm, 9 µm particle size; Aminex HPX-87H; Bio-Rad; Hercules, CA, USA). The mobile phase was 0.5 mM H_2_SO_4_ and the flow rate was 0.6 mL/min for 23 min. The temperature of the column and detector cell were maintained at 65 ℃ and 35 ℃, respectively [51].

Because 1,2-PDO and 1,3-PDO have the same HPLC retention times (16.8 min), gas chromatography (GC) was used with HPLC to analyze these compounds. The GC system (Agilent Technologies 7890A, Agilent Technologies, Santa Clara, CA, USA) had a flame ionization detector and an ZB-WAXplus column (30 m × 0.25 mm, df = 0.25 µm; Zebron Phenomenex), and nitrogen was the carrier gas. The injector and detector were maintained at 250 ℃ and 280 ℃, respectively. The column temperature was 120 ℃ for 1 min, increased at a rate of 20 ℃/min to 230 ℃, and was then maintained at 230 °C for 1.3 min [15]. All experiments were performed (biological and experimental) in triplicate.

## Supplementary Information

**Additional file 1**: **Figure. S1**. The appearance of a 5-L fermenter at agitation speed of 500 rpm. **Figure. S2**. Schematic representation of plasmid pBR-1,2PDO construction. **Figure. S3**. Construction of the *adhE*-deficient mutant of *K. pneumoniae *GEM167 by substitution of *adhE* with an apramycin resistance gene [*aac(3)IV*] via homologous recombination. **Figure. S4**. Construction of the *budA*-deficient mutant of *K. pneumoniae* GEM167*ΔadhE* by substitution of *budA* with an apramycin resistance gene [*aac(3)IV*] via homologous recombination. **Table S1**. Oligonucleotide primers used in this study.

## Data Availability

Not applicable.

## References

[1] Yang F, Hanna MA, Sun RJB (2012). Value-added uses for crude glycerol–a byproduct of biodiesel production. Biotechnol Biofuels.

[2] Da Silva GP, Mack M, Contiero JJB (2009). Glycerol: a promising and abundant carbon source for industrial microbiology. Biotechnol Adv.

[3] Dharmadi Y, Murarka A, Gonzalez R (2006). Anaerobic fermentation of glycerol by *Escherichia coli*: a new platform for metabolic engineering. J Biotechnol Bioeng.

[4] Kumar V, Park S (2018). Potential and limitations of Klebsiella pneumoniae as a microbial cell factory utilizing glycerol as the carbon source. J Biotechnol Adv.

[5] Yazdani SS, Gonzalez R (2007). Anaerobic fermentation of glycerol: a path to economic viability for the biofuels industry. J Current Opinion Biotechnol.

[6] Shelley S (2007). A renewable route to propylene glycol. J Chem Eng Prog.

[7] Martin AE, Murphy FH (2000). Glycols, propylene glycols J Kirk-Othmer Encyclopedia of Chemical Technology.

[8] Ahorsu R, Medina F, Constantí M (2018). Significance and challenges of biomass as a suitable feedstock for bioenergy and biochemical production: a review. J Energies.

[9] Suzuki T, Onishi H (1968). Aerobic dissimilation of L-rhamnose and the production of L-rhamnonic acid and 1, 2-propanediol by yeasts. J Agricultural Biological Chem.

[10] Weimer PJ (1984). Fermentation of 6-deoxyhexoses by Bacillus macerans. J Appl Environ Microbiol.

[11] Tran-Din K, Gottschalk G (1985). Formation of D (-)-1, 2-propanediol and D (-)-lactate from glucose by *Clostridium sphenoides* under phosphate limitation. J Archives Microbiol.

[12] Cameron DC, Cooney CL (1986). A novel fermentation: the production of R (–)–1, 2–propanediol and acetol by *Clostridium thermosaccharolyticum*. Bio/Technology.

[13] Bennett G, San K-Y (2001). Microbial formation, biotechnological production and applications of 1, 2-propanediol. J Appl Microbiol Biotechnol.

[14] Zeng A-P, Sabra W (2011). Microbial production of diols as platform chemicals: recent progresses. J Current Opinion Biotechnol.

[15] Sun S, Shu L, Lu X, Wang Q, Tišma M, Zhu C (2021). 1, 2-Propanediol production from glycerol via an endogenous pathway of *Klebsiella pneumoniae*. Appl Microbiol Biotechnol.

[16] Ferguson GP, Chacko AD, Lee C, Booth IR, Lee C (1996). The activity of the high-affinity K+ uptake system Kdp sensitizes cells of *Escherichia coli* to methylglyoxal. J Bacteriol.

[17] Booth IR, Ferguson G, Miller S, Li C, Gunasekera B, Kinghorn S (2003). Bacterial production of methylglyoxal: a survival strategy or death by misadventure?. J Biochem Soc Trans.

[18] Niu W, Guo J (2015). Stereospecific microbial conversion of lactic acid into 1, 2-propanediol. J ACS Synthetic Biol.

[19] Niu W, Kramer L, Mueller J, Liu K, Guo J (2019). Metabolic engineering of *Escherichia coli* for the de novo stereospecific biosynthesis of 1, 2-propanediol through lactic acid. Metabolic Eng Communications.

[20] Arasu MV, Kumar V, Ashok S, Song H, Rathnasingh C, Lee HJ (2011). Isolation and characterization of the new *Klebsiella pneumoniae* J2B strain showing improved growth characteristics with reduced lipopolysaccharide formation. Biotechnol Bioproc Eng.

[21] Kumar V, Sankaranarayanan M, Jae K-e, Durgapal M, Ashok S, Ko Y (2012). Co-production of 3-hydroxypropionic acid and 1, 3-propanediol from glycerol using resting cells of recombinant *Klebsiella pneumoniae* J2B strain overexpressing aldehyde dehydrogenase. Appl Microbiol Biotechnol.

[22] Celińska E (2012). Klebsiella spp as a 1, 3-propanediol producer–the metabolic engineering approach. J Critical Rev Biotechnol.

[23] Kumar V, Ashok S, Park SJB (2013). Recent advances in biological production of 3-hydroxypropionic acid. Biotechnol Adv.

[24] Oh B-R, Seo J-W, Heo S-Y, Hong W-K, Luo LH, Joe M-h (2011). Efficient production of ethanol from crude glycerol by a *Klebsiella pneumoniae* mutant strain. Bioresource Technol.

[25] Badía J, Ros J, Aguilar J (1985). Fermentation mechanism of fucose and rhamnose in *Salmonella typhimurium* and *Klebsiella pneumoniae*. J Bacteriol.

[26] Sanchez-Riera F, Cameron D, Cooney C (1987). Influence of environmental factors in the production of R (−)-1, 2-propanediol by *Clostridium thermosaccharolyticum*. Biotech Lett.

[27] Tao Y-m, Bu C-y, Zou L-h, Hu Y-l, Zheng Z-J, Ouyang J (2021). A comprehensive review on microbial production of 1, 2-propanediol: micro-organisms, metabolic pathways, and metabolic engineering. J Biotechnol Biofuels.

[28] Tsvetanova F, Petrova P, Petrov K (2014). 2, 3-Butanediol production from starch by engineered *Klebsiella pneumoniae* G31-A. J Appl Microbiol Biotechnol.

[29] Petrov K, Petrova P (2009). High production of 2, 3-butanediol from glycerol by *Klebsiella pneumoniae* G31. J Appl Microbiol Biotechnol.

[30] Cheng K-K, Liu D-H, Sun Y, Liu W-B (2004). 1, 3-Propanediol production by *Klebsiella pneumoniae* under different aeration strategies. J Biotechnol letters.

[31] Oh BR, Lee SM, Heo SY, Seo JW, Kim CH (2018). Efficient production of 1,3-propanediol from crude glycerol by repeated fed-batch fermentation strategy of a lactate and 2,3-butanediol deficient mutant of Klebsiella pneumoniae. Microb Cell Fact.

[32] Celińska E, Grajek W (2009). Biotechnological production of 2, 3-butanediol—current state and prospects. J Biotechnol Adv.

[33] Liu L, Chen J (2011). Cofactor engineering enhances the physiological function of an industrial strain. J Progress Mol Environ Bioeng Anal Model Technol Appl.

[34] Foster JW, Park Y, Penfound T, Fenger T, Spector M (1990). Regulation of NAD metabolism in *Salmonella typhimurium*: molecular sequence analysis of the bifunctional nadR regulator and the nadA-pnuC operon. J bacteriol.

[35] Heux S, Cachon R, Dequin S (2006). Cofactor engineering in *Saccharomyces cerevisiae*: expression of a H2O-forming NADH oxidase and impact on redox metabolism. J Metabolic Eng.

[36] Ji X-J, Xia Z-F, Fu N-H, Nie Z-K, Shen M-Q, Tian Q-Q (2013). Cofactor engineering through heterologous expression of an NADH oxidase and its impact on metabolic flux redistribution in *Klebsiella pneumoniae*. J Biotechnol Biofuels.

[37] Zhang Y, Li Y, Du C, Liu M, Za C (2006). Inactivation of aldehyde dehydrogenase: a key factor for engineering 1,3-propanediol production by *Klebsiella pneumoniae*. Metabolic Eng.

[38] Zeng A-P, Ross A, Biebl H, Tag C, Gunzel B, Deckwer W-D (1994). Multiple product inhibition and growth modeling of *Clostridium butyricum* and *Klebsiella pneumoniae* in glycerol fermentation. Biotechnol Bioeng.

[39] Ji X-J, Huang H, Ouyang P-K (2011). Microbial 2,3-butanediol production: a state-of-the-art review. Biotechnol Adv.

[40] Zhang G, Yang G, Wang X, Guo Q, Li Y, Li J (2012). Influence of blocking of 2,3-butanediol pathway on glycerol metabolism for 1,3-propanediol production by *Klebsiella pneumoniae*. Appl Biochem Biotechnol.

[41] Zhu Y, Yang S-T (2004). Effect of pH on metabolic pathway shift in fermentation of xylose by *Clostridium tyrobutyricum*. J Biotechnol.

[42] Berrıos-Rivera SJ, Bennett GN, San K-Y (2002). Metabolic engineering of *Escherichia coli*: increase of NADH availability by overexpressing an NAD+-dependent formate dehydrogenase. Metab Eng.

[43] Zhang G, Ma B, Xu X, Li C, Wang L (2007). Fast conversion of glycerol to 1,3-propanediol by a new strain of *Klebsiella pneumoniae*. Biochem Eng J.

[44] Oh B-R, Heo S-Y, Lee S-M, Hong W-K, Park JM, Jung YR (2014). Production of 2-butanol from crude glycerol by a genetically-engineered *Klebsiella pneumoniae* strain. Biotechnol Lett.

[45] Datsenko KA, Wanner BL (2000). One-step inactivation of chromosomal genes in *Escherichia coli* K-12 using PCR products. J Proc Natl Acad Sci.

[46] Cherepanov PP, Wackernagel WJG (1995). Gene disruption in *Escherichia coli*: TcR and KmR cassettes with the option of Flp-catalyzed excision of the antibiotic-resistance determinant. Gene.

[47] Mu Y, Teng H, Zhang DJ, Wang W, Xiu ZL (2006). Microbial production of 1,3-propanediol by *Klebsiella pneumoniae* using crude glycerol from biodiesel preparations. Biotechnol Lett.

[48] Fournet-Fayard S, Joly B, Forestier C (1995). Transformation of wild type *Klebsiella pneumoniae* with plasmid DNA by electroporation. J Microbiol Methods.

[49] Seo M-Y, Seo J-W, Heo S-Y, Baek J-O, Rairakhwada D, Oh B-R (2009). Elimination of by-product formation during production of 1,3-propanediol in *Klebsiella pneumoniae* by inactivation of glycerol oxidative pathway. Appl Microbiol Biotechnol.

[50] Oh B-R, Hong W-K, Heo S-Y, Joe M-h, Seo J-W, Kim CH (2013). The role of aldehyde/alcohol dehydrogenase (AdhE) in ethanol production from glycerol by *Klebsiella pneumoniae*. J Ind Microbiol Biotechnol.

[51] Ju J-H, Heo S-Y, Choi S-W, Kim Y-M, Kim M-S, Kim C-H (2021). Effective bioconversion of 1,3-propanediol from biodiesel-derived crude glycerol using organic acid resistance-enhanced *Lactobacillus reuteri* JH83. Bioresour Technol.

