# Supplementary material for: Production of 1,2-propanediol from glycerol in Klebsiella pneumoniae GEM167 with flux enhancement of the oxidative pathway
Source: Biotechnol Biofuels Bioprod. 2023 Feb 6;16:18. doi: 10.1186/s13068-023-02269-4 (PMC9903448; doi:10.1186/s13068-023-02269-4)
Supplement: Supplementary file 1 — Additional file 1: Figure. S1. The appearance of a 5-L fermenter at agitation speed of 500 rpm. Figure. S2. Schematic representation of plasmid pBR-1,2PDO construction. Figure. S3. Construction of the adhE-deficient mutant of K. pneumoniae GEM167 by substitution of adhE with an apramycin resistance gene [aac(3)IV] via homologous recombination. Figure. S4. Construction of the budA-deficient mutant of K. pneumoniae GEM167ΔadhE by substitution of budA with an apramycin resistance gene [aac(3)IV] via homologous recombination. Table S1. Oligonucleotide primers used in this study. [file 13068_2023_2269_MOESM1_ESM.docx]

**Supplementary information**

**Production of 1,2-propanediol from glycerol in *Klebsiella pneumoniae* GEM167 with flux enhancement of the oxidative pathway**

Min-Ho Jo ^a^, Sun-Yeon Heo^a^, Jung-Hyun Ju^a^, Jaehoon Cho^b^, Ki Jun Jeong^c^, Min-Soo Kim^a^, Chul-Ho Kim^a^ and Baek-Rock Oh^a,*^

^a^Microbial Biotechnology Research Center, Jeonbuk Branch Institute, Korea Research Institute of Bioscience and Biotechnology (KRIBB), Jeongeup, Jeonbuk 56212, Republic of Korea.

^b^Green and Sustainable Materials R&D Department, Korea Institute of Industrial Technology, Cheonan, Chungcheongnam 31056, Republic of Korea.

^c^Department of Chemical and Biomolecular Engineering and Institute for the BioCentury, KAIST, Daejeon 34141, Republic of Korea

*** Correspondence:**

Baek-Rock Oh, Microbial Biotechnology Research Center, Jeonbuk Branch Institute, Korea Research Institute of Bioscience and Biotechnology (KRIBB), Jeongeup, Jeonbuk 56212, Republic of Korea. Tel: +82 63 570 5117; Fax: +82 63 570 5160; E-mail: baekrock.oh@kribb.re.kr (B. R. Oh)

**
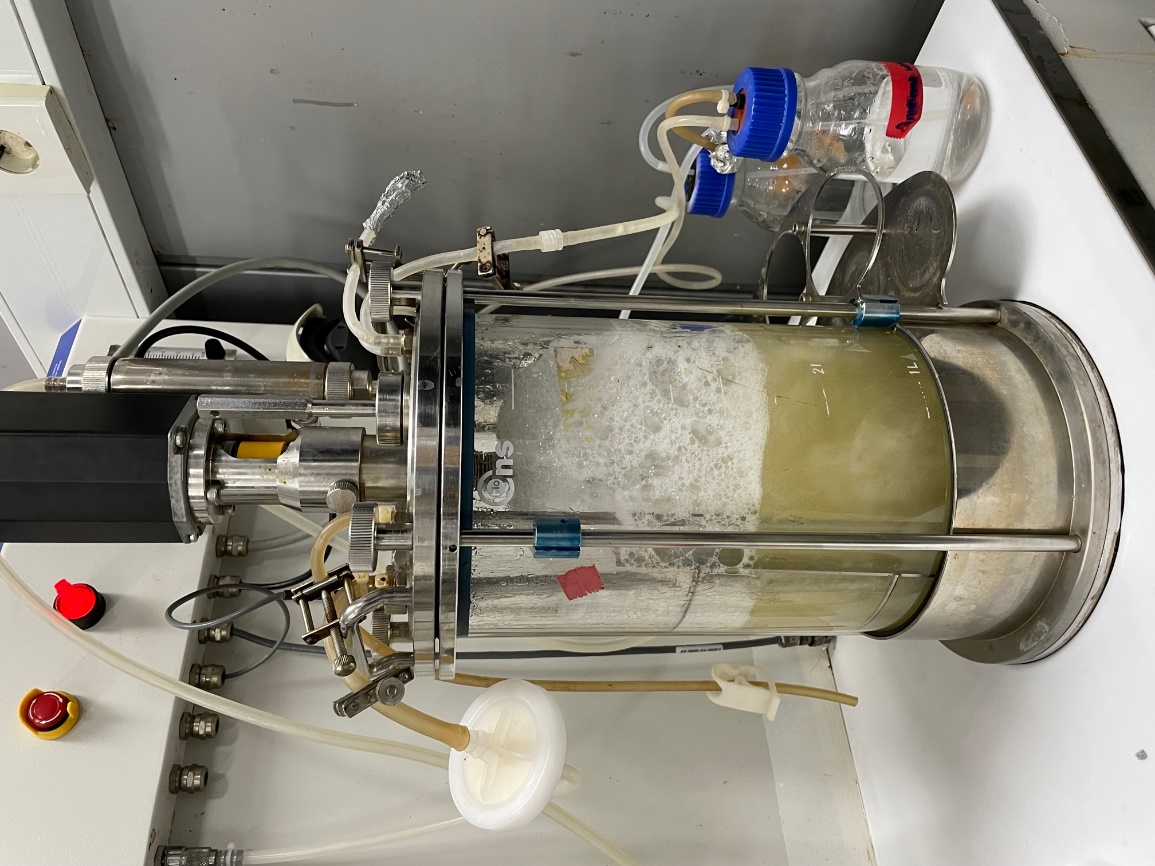
**

**Fig. S1.** The appearance of a 5L-fermentor at agitation speed of 500 rpm.


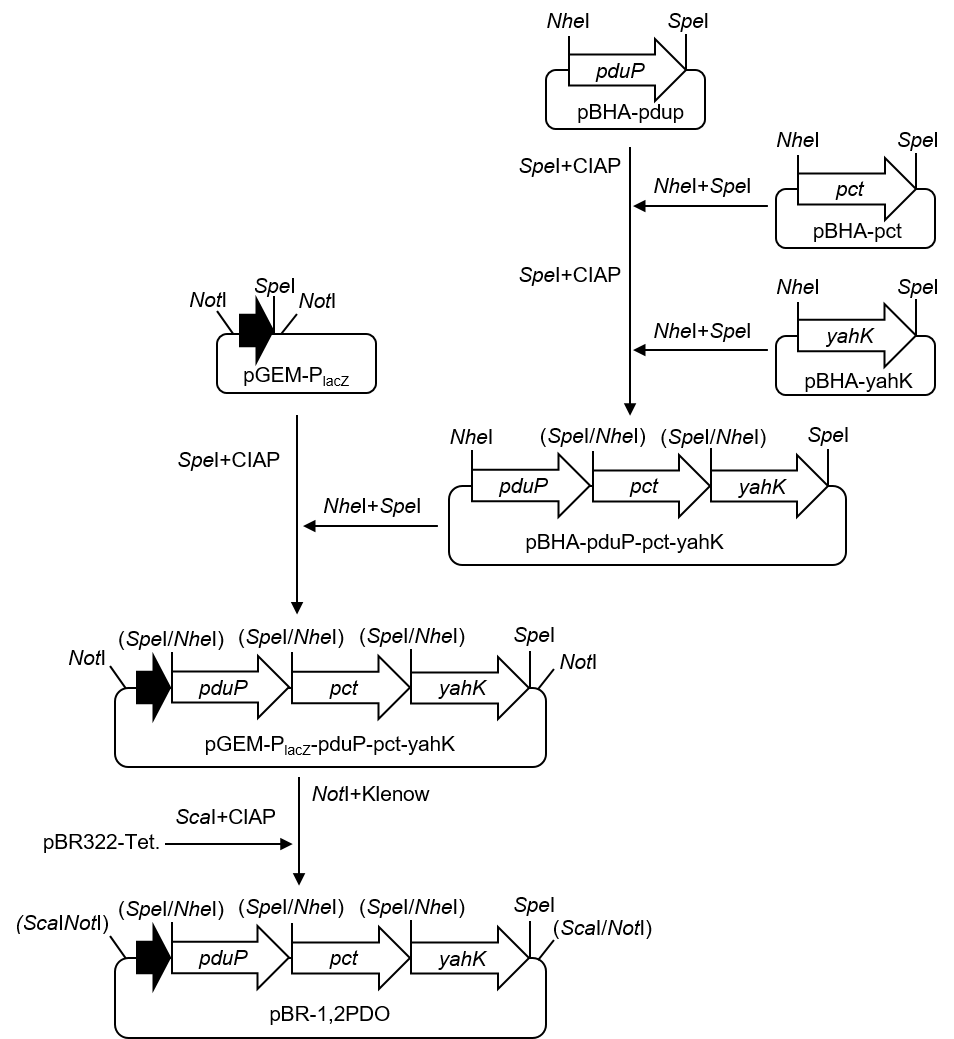


**Fig. S2.** Schematic representation of plasmid pBR-1,2PDO construction.

**
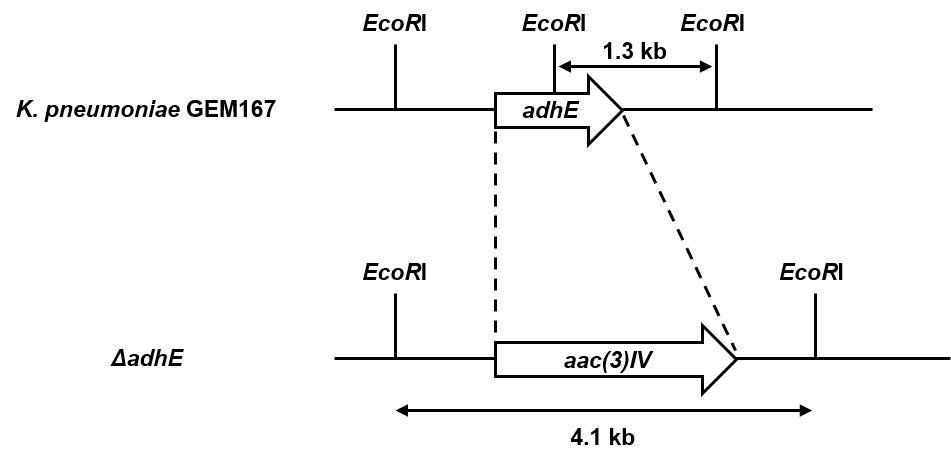
**

**Fig. S3.** Construction of the *adhE*-deficient mutant of *K. pneumoniae* GEM167 by substitution of *adhE* with an apramycin-resistance gene [*aac(3)IV*] via homologous recombination.

**
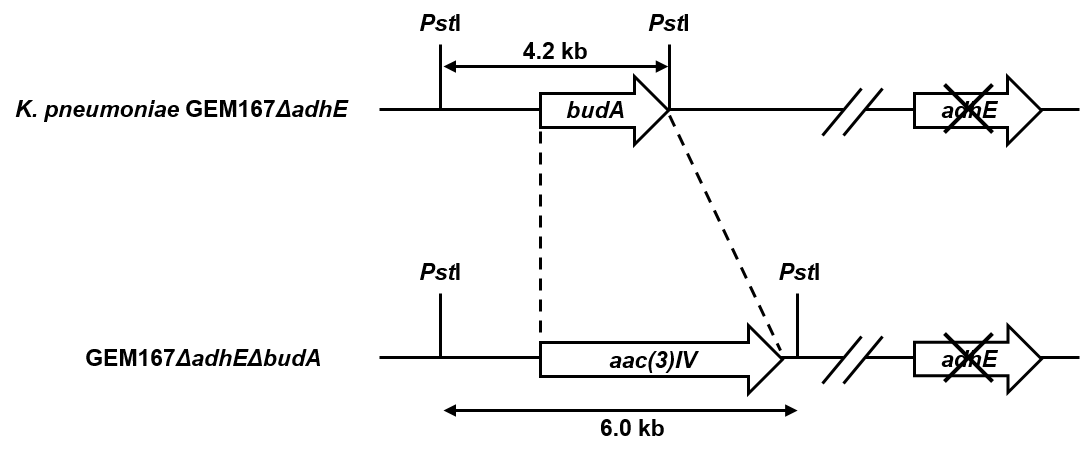
**

**Fig. S4.** Construction of the *budA*-deficient mutant of *K. pneumoniae* GEM167*ΔadhE* by substitution of *budA* with an apramycin-resistance gene [*aac(3)IV*] via homologous recombination.

**Table S1.** Oligonucleotide primers used in this study

| Primer | Sequence (5’-3’) |
| --- | --- |
| *pct-F* | ATGCGCAAGGTTGAGATTAT |
| *pct-R* | TTACTTTTTCAACCCCATTG |
| *pduP-F* | ATGAATACTTCTGAATTGGA |
| *pduP-R* | TTATCTAATAGAAAAGCCGT |
| *yahK-F* | ATGAAGATCAAGGCTGTTGG |
| *yahK-R* | TTAGTCTGTTAGAGTGCGAT |

*pct*: lactoyl-CoA transferase; propionate-CoA transferase from *Megasphaera elsdenii*

*pduP*: CoA-dependent lactaldehyde dehydrogenase; CoA-dependent propanal dehydrogenase from *Salmonella enterica*

*yahK*: lactaldehyde reductase; aldehyde reductase from *E. coli*
